# miR-133a silencing rescues glucocorticoid-induced bone loss by regulating the MAPK/ERK signaling pathway

**DOI:** 10.1186/s13287-021-02278-w

**Published:** 2021-03-29

**Authors:** Gang Wang, Fengbin Wang, Lecheng Zhang, Chao Yan, Yuelei Zhang

**Affiliations:** Department of Orthopedics, the First Affiliated Hospital of Anhui Medical University, 218 Jixi Road, Hefei, 230000 China

**Keywords:** Mesenchymal stem cells, Glucocorticoid-induced osteoporosis, microRNA-133a, FGFR1, ERK

## Abstract

**Background:**

Dysfunction of mesenchymal stem cells (MSCs) is recognized as critical to the pathogenesis of glucocorticoid-induced osteoporosis (GIO), suggesting the potential of MSC-targeting interventions for this disorder. As the miR-133a has been shown to play an important role in bone metabolism, we hypothesized that miR-133a may also be involved in GIO.

**Methods:**

In the in vitro study, we examined the effect of miR-133a antagomir on DEX-treated MSCs, including proliferation, apoptosis, osteoblast, and adipocyte differentiation, then, we explored the mechanism of these effects of miR-133a silencing through measuring the phosphorylation of ERK1/2 and its regulator FGFR1 via western blot and qRT-PCR. In the in vivo study, we developed a GIO rat model by injecting methylprednisolone and modulated the miR-133a expression in the femur by intramedullary injection of the miR-133a antagomir, and then micro-CT analyses and histological staining of the femurs were used to investigate the effect of miR-133a silencing on bone loss of the GIO rats.

**Results:**

qRT-PCR analysis indicated that glucocorticoid induced high miR-133a expression in MSCs and animal models. The in vitro study showed that miR-133a antagomir significantly promoted cell proliferation, viability, and osteoblast differentiation and inhibited adipocyte differentiation in DEX-treated MSCs. Furthermore, the expression of p-ERK1/2 and FGFR1 in DEX-treated MSCs was also upregulated by miR-133a antagomir. Then we investigated the effect of miR-133a silencing on the bone architecture of GIO models, micro-CT analysis showed that miR-133a antagomir attenuated the loss of bone mass and improved the trabecular and cortical parameters induced by methylprednisolone. Histological study showed that miR-133a silencing simultaneously increased bone formation and decreased marrow fat accumulation in GIO rats.

**Conclusions:**

Our findings suggested that miR-133a is strongly associated with GIO and similar disorders induced by glucocorticoids in MSCs. Silencing miR-133a resulted in positive effects on GC-treated MSCs and on bone loss in GIO animal models. Moreover, the FGFR1-MAPK/ERK signaling may be involved in the protective effect of miR-133a silencing.

## Background

Long-term exposure to glucocorticoids (GCs) at pharmacological dosages is the most common cause of secondary osteoporosis. Dysfunction of mesenchymal stem cells (MSCs), the progenitors of bone cells, has been recognized as critical to the pathogenesis [1]. Investigations into the pathogenic mechanisms have shown that glucocorticoid inhibits telomerase activity in MSC accelerates their aging and attenuates their ability for self-renewal, resulting in an inhibition of MSC proliferation [2]. Moreover, GCs are antagonists of Runx2, a key regulator of osteoblast differentiation, thus reducing the activity of Runx2 protein and inhibiting MSC differentiation into osteoblasts [3]. GCs also upregulate the expression of adipogenesis-associated proteins, such as peroxisome proliferator-activated receptor γ (PPARγ) and adipocyte protein 2 (aP2) [4], altering the balance between osteogenesis and adipogenesis and promoting differentiation of MSCs into adipocytes [5]. Therefore, investigations into GC-induced osteoporosis (GIO) have focused on MSC interventions [6–8].

MicroRNAs are endogenous single-stranded non-coding RNAs that regulate a variety of biological processes by binding to mRNA [8]. Recently, several microRNAs have been reported to be involved in osteoporosis, such as miR-106b [8], miR23a/b [9], and miR-188 [10]. Interestingly, miR-133a, a highly conserved microRNA that plays a critical role in skeletal and cardiac muscle [11], was also revealed to be very important in bone metabolism. The miRNA profile showed that miR-133a was downregulated during BMP2-mediated mesenchymal cell osteogenic differentiation and further study indicated that miR-133a inhibits osteogenesis by directly targeting Runx2 [12]. Other studies have reported that miRNA-133a was upregulated in ovariectomized mice [13] and postmenopausal osteoporotic patients [14] while localized inhibition of miR-133a enhanced MSC-mediated osteogenesis [15]. All these studies suggest that miR-133a may be a key target in the treatment of osteoporosis.

In this study, we further explored the role of miR-133a in GIO and discovered that miR-133a was closely related to glucocorticoid-induced MSC disorders and subsequent bone loss. Silencing of miR-133a exerted a positive effect on GC-treated MSCs and prevented bone loss in rats with GIO. We further demonstrated that the MAPK/ERK signaling pathway was involved in this process and may be a target of miR-133a.

## Materials and methods

### Cell culture and transfection

Primary human bone marrow-derived MSCs were purchased from the ATCC (Manassas, VA, USA) and cultured in DMEM culture medium (Hyclone, Logan, UT, USA) containing 10% fetal bovine serum (Secure, New Zealand, Gibco, Thermo Fisher, Waltham, MA, USA) plus 1% penicillin/ streptomycin (Gibco) at 37 °C with 5% CO_2_. Cells were passaged at 80–90% confluence, and MSCs after three to six passages were used in this study. To test the effects of glucocorticoid treatment, MSCs were incubated in the presence of 10^− 5^ M dexamethasone (DEX; Sigma. St Louis, MS, USA) for 48 h.

To downregulate the expression of miR-133a in MSCs, cells were transfected with a miR-133a antagomir and its negative control (RIBOBIO, Guangzhou, China) according to the manufacturer’s instructions prior to the following studies.

### Cell proliferation and apoptosis

To explore the effect of miR-133a silencing on the proliferation of GC-treated MSCs, the Cell Counting Kit-8 (CCK-8, Beyotime Biotechnology, Shanghai, China) assay was performed following the manufacturer’s instructions. Specifically, MSCs with or without miR-133a silence were incubated in 96-well plates with 3 wells in each group at an initial density of 5 × 10^3^ cells/well. Then the culture medium was discarded and 100 μL culture medium plus 10 μL CCK-8 were added after 24 h (day 1), 48 h (day 2), 72 h (day 3), 96 h (day 4), and 120 h (day 5) respectively. After incubation for 2 h, the 450 nm absorbance values for each well were measured in a microplate (Bio-Rad, Hercules, CA) reader. The ratio of absorbance relative to the absorbance on day 1 was used as a measure of the cell proliferation rate.

The Annexin V-FITC cell apoptosis detection kit (AAT Bioquest, USA) was used to investigate the effect of miR-133a silencing on MSC apoptosis. Cells were cultured in FBS-free medium for 48 h followed by resuspension in 200 μL Annexin V-FITC and 10 μL propidium iodide. The apoptotic cells were Annexin V-positive and PI-negative. Cells were analyzed with flow cytometry, and the ratio of apoptotic cells relative to total cells was measured.

### Osteoblast and adipocyte differentiation

To induce differentiation into osteoblasts or adipocytes, MSCs were cultured in 6-well plates at an initial density of 1 × 10^6^ cells/well, after propagating to a density of more than 80%, MSCs were incubated with osteogenesis induction medium (full medium, 300 ng/ml BMP-2, 50 μg/ml ascorbic acid, and 5 mM β-glycerophosphate) or adipogenesis induction medium (full medium, 0.5 mM 3-isobutyl-1-methylxanthine, 10 μg/ml insulin, and 200 μM indometacin), as described previously [10], for 48 h. The cells were then collected for subsequent western blot and qRT-PCR analysis.

To further investigate the mineralization capacity, MSCs were cultured with osteogenesis induction medium for 21 days, stained with 2% Alizarin Red S and the cell matrix mineralization evaluated with an inverted microscope (Leica, German). Quantification was performed using Image Pro Plus 6.0 software from at least five randomly selected microscopic fields, the relative area of mineralized matrix was calculated as the area of mineralized matrix/total area.

After incubation with adipogenesis induction medium for 14 days, oil red staining was performed to evaluate the presence of mature adipocytes in cultures. Similar to alizarin red staining, quantification was performed using Image Pro Plus 6.0 software from at least five randomly selected microscopic fields, and the relative area of adipocytes was calculated as the area of adipocytes /total area.

### Western blotting

Western blotting analysis for osteogenesis- or adipogenesis-related proteins was performed as described previously [7]. The total protein was extracted and quantified with a BCA kit (Beyotime Biotechnology, Shanghai, China), after which proteins (60 μg total protein per sample) were separated on SDS-PAGE and transferred to a PVDF membrane. The membrane was blocked with 5% BSA for 1 h at room temperature and incubated with specific antibodies to RunX2 (Abcam, 1:1000), ALP (Abcam, 1:500), ERK1/2 (CST, 1:1000), p-ERK1/2(CST, 1:1000), ap2 (Abcam, 1:2000), PPARγ (Abcam, 1:800), FGFR1(Abcam, 1:1000), and GAPDH (Abcam, 1:1000) at 4 °C overnight. After probing with the appropriate HRP conjugated secondary antibodies, and chemiluminescence using ECL assay (Thermo Pierce, USA), the membrane was exposed to X-ray film for 5 min. The target bands were quantified using Quantity ONE software (Bio-Rad, Hercules, CA, USA) and normalized to GAPDH.

### qRT-PCR analysis

To investigate the level of osteogenesis- or adipogenesis-related mRNAs in the samples, total RNA was extracted using the TRIzol reagent (Invitrogen, Thermo Fisher, Waltham, MA, USA) and reversely transcribed using SuperScript™ III First-Strand Synthesis SuperMix for qRT-PCR (Invitrogen). Amplification reactions were set up in a 20 μL volume containing 1 μL cDNA, Power SYBR Green Master Mix, and amplification primers. Gene expression was normalized to β-actin. The primer sequences are listed in the Table 1.

**Table 1:** Primer sequences in the quantitative real-time polymerase chain reaction analysis of osteogenesis- and adipogenesis-related mRNAs

Gene	Forward primer	Reverse primer
*Runx2*	GCCTCTTCAGCACAGTGACA	CATTCCGGAGCTCAGCAGAA
*ALP*	ACCGCTTCCCATATGTGGCT	TGCACCAGATTCTTCCCGTC
*aP2*	TGACAGGAAAGTCAAGAGCACC	GCCTTTCATGACGCATTCCA
*PPARγ*	CTTGCAGTGGGGATGTCTCAT	AGCAAACCTGGGCGGTTGAT
*FGFR1*	TCAGATGCTCTCCCCTCCTC	CTACGGGCATACGGTTTGGT
*GAPDH*	CCATGACAACTTTGGTATCGTGGAA	GGCCATCACGCCACAGTTTC

To investigate the expression of miR-133a, microRNA was extracted using the PureLink miRNA Isolation Kit (Thermo Fisher) and reversely transcribed using SuperScript III Reverse Transcriptase (Invitrogen) containing the stem-loop RT primer for miR-133a and U6. Then, 1 μL cDNA was amplified in a 20 μL volume containing Power SYBR Green Master Mix and specific amplification primers. The step-loop RT primer for miR-133a was 5′-GTCGTATCCAGTGCAGGGTCCGAGGTA TTCGCACTGGATACGACCAGCTG-3′, the forward primer for qRT-PCR was 5′-GCGTTTGGTCCCCTTCAACCA-3′ and the reverse primer was 5′-AGTGCAGGGTCCGAGGTATT-3′. miR-133a expression was normalized to U6.

### Animal models and treatment

In this study, 40 female Sprague-Dawley rats were obtained from the Animal Center of Anhui Medical University and divided equally into four groups: the Blank group that received normal feed and subsequent 40 μL PBS injection into medullary cavity, the methylprednisolone (MP) group that received a 20 mg/kg BW methylprednisolone (40 mg/ml) intramuscular injection on three consecutive days a week for 3 weeks (nine injections in all), as previously described [2], and a subsequent PBS intramedullary injection, the MP + antagomir negative control (NC) group that received intramuscular methylprednisolone injections and 40 μL miR-133a antagomir NC femoral intramedullary injections every 2 weeks, and the MP + antagomir-133a group that received intramuscular methylprednisolone injections and 40 μL miR-133a antagomir femoral intramedullary injections every 2 weeks. The intramedullary injection followed the first methylprednisolone injection for local miR-133a silencing to avoid the effects of intravenous injection and was performed with a microinjector into the bilateral proximal femur. Samples were collected 8 weeks after the first methylprednisolone injection. All animal experiments were approved by the Institutional Animal Care and Use Committee of Anhui Medical University, and all animals were housed in the Centers for Laboratory Animal Care at the Anhui Medical University.

### Micro-CT analysis

To evaluate the bone architectural changes in the rats with different treatments, the left total femur of each rat was scanned at a voxel of 12 μm. Cross-sectional images were transferred to CTAn software (Bruker, Billerica, MA, USA) to analyze the trabecular parameters and then reconstructed with CTVox (Bruker). For the distal femur, the region of interest (ROI) selected for analysis was 3.6 mm femoral length above the growth plate; while for the femoral head, the ROI started from 0.54 to 1.72 mm below the top of the femoral head. Trabecular parameters were as follows: bone mineral density (BMD), bone volume per tissue volume (BV/TV), trabecular thickness (Tb. Th), trabecular number (Tb. N), and trabecular separation (Tb. Sp). In addition, cortical thickness (Ct. Th) was used to evaluate the middle femur cortex and the ROI started from 12.6 to 13.5 mm above the growth plate.

### Histological analysis

The distal and proximal femurs were decalcified and embedded in paraffin, following which 5-μm sections were cut and stained with H&E to evaluate the trabecular morphological changes and adipocytes in the bone marrow. Photomicrographs were obtained with a LEICA DM 4000. Number of adipocytes in the bone marrow were counted from five randomly selected 400-fold microscopic fields and calculated as number of adipocytes/total area.

Immunohistochemical analysis was performed as described previously [2]. Specifically, femur sections were deparaffinized, the antigen retrieved and then incubated with anti-osteocalcin (OCN) primary antibody (Affinity, USA) at 4 °C overnight. After incubation with the biotinylated secondary antibody, the protein in the sections was visualized with 3,3′-diaminobenzidine and counterstained with hematoxylin. The number of OCN-positive osteoblasts was counted from five randomly selected 400-fold microscopic fields and calculated as number of osteoblasts/bone surface.

Immunofluorescence was performed to evaluate the expression of perilipin, a lipid droplet-associated protein. In brief, sections were deparaffinized and the antigens retrieved. Sections were then blocked with 5% BSA and probed with rabbit anti-perilipin antibody (Affinity, USA) overnight at 4 °C, before incubation with an anti-rabbit fluorescence-labeled secondary antibody (CST, USA). Digital images were scanned with an Olympus FV1200 confocal microscope. The perilipin-positive area from five randomly selected 400-fold microscopic fields was quantitatively measured with the Image Pro Plus 6.0 software, and the relative expression of perilipin was calculated as the area of perilipin /total area.

### Statistical analysis

The values from each sample were analyzed with the SPSS software package (Version 17.0, Microsoft, Chicago, IL, USA). Values were analyzed with one-way analysis of variance with Tukey’s post hoc test. Differences were considered to be statistically significant when the *P* value was less than 0.05.

## Results

### Glucocorticoid induces high miR-133a expression in MSCs and animal models

**Fig. 1:**
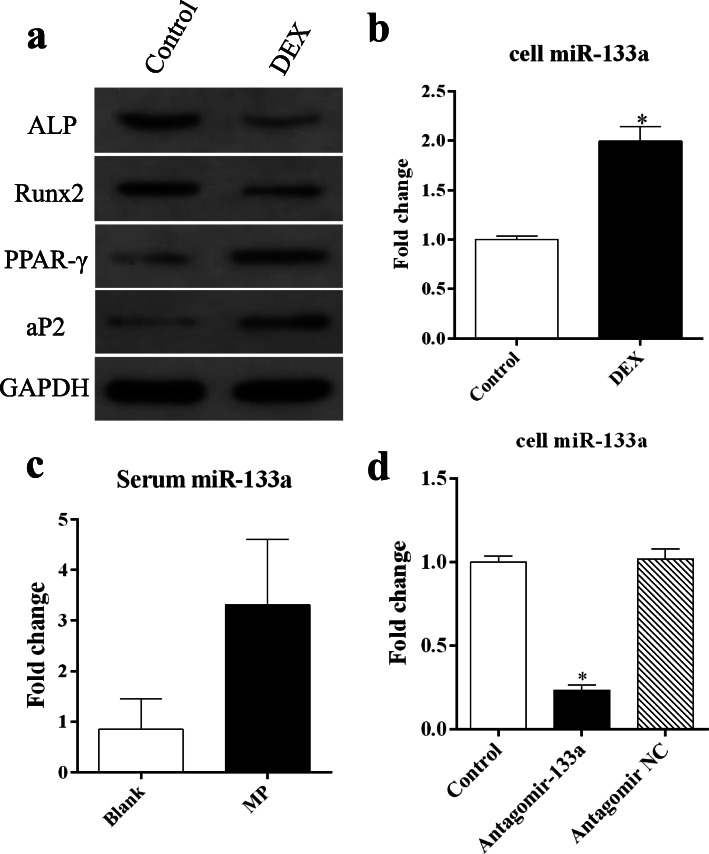
miR-133a expression in glucocorticoid-treated MSCs and animal models. **a** Western blotting analysis for osteogenesis and adipogenesis-related proteins in MSCs after treatment with DEX. **b** Relative levels of miR-133a in MSCs after DEX treatment. **c** Relative levels of miR-133a in the serum of rats after methylprednisolone intramuscular injection. **d** Relative levels of miR-133a in MSCs transfected with the miR-133a antagomir (each condition was performed in triplicate, *P < 0.05 relative to the control or blank group)

We used in vivo and in vitro experiments to investigate whether miR-133a is involved in the pathogenesis of GIO. In vitro, DEX significantly downregulated the levels of the osteogenesis-related proteins Runx2 and ALP while upregulating the expression of the adipogenesis-related proteins aP2 and PPARγ (Fig. 1a), in agreement with previous studies [3–5]. Notably, the miR-133a level was upregulated accompanied by significant damage to the MSC differentiation process by DEX (Fig. 1b). Serum miR-133a was consistently upregulated in MP-treated rats (Fig. 1, c), suggesting that miR-133a may negatively modulate the development of GIO. Thus, we speculated that silencing miR-133a (Fig. 1d) may attenuate the side effects of the glucocorticoid.

### Silencing miR-133a in MSCs promoted cell proliferation and cell viability

**Fig. 2:**
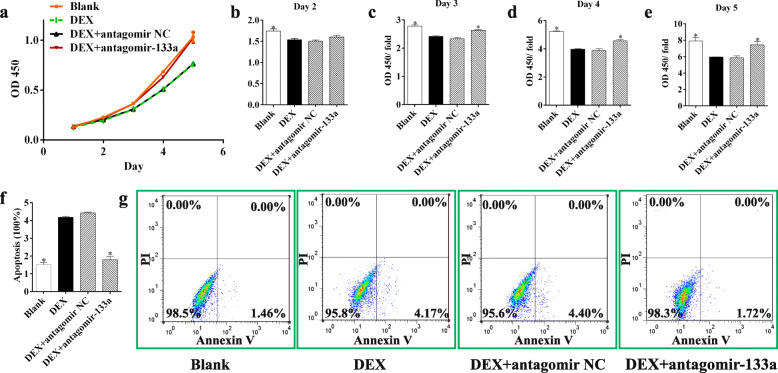
Silencing miR-133a in MSCs promoted cell proliferation and cell viability. **a** CCK-8 assay for MSC proliferation. **b–e** Increase in MSC proliferation from day 2 to day 5 relative to day 1 with initial cell attachment. **f**, **g** The flow cytometric analysis for Annexin V-positive and PI-negative apoptotic MSCs (each condition was performed in triplicate, * *P* < 0.05 relative to the DEX group)

To investigate the effect of miR-133a silencing on GC-treated MSCs, two groups of cells were transfected with the miR-133a antagomir and its negative control, respectively, and then cultured with DEX. The CCK-8 assay showed that MSCs normally proliferated rapidly from day 3 which was significantly suppressed by DEX (Fig. 2a). Silencing miR-133a in MSCs attenuated the DEX suppression, particularly after 3 days’ incubation (Fig. 2b–e).

We then explored the effect of miR-133a silencing on the viability of GC-treated MSCs. Apoptosis was induced by culture with FBS-free medium and the Annexin V-positive/PI-negative apoptotic cells were analyzed with flow cytometry. The results showed that after FBS-free culture for 48 h, DEX treatment resulted in increased numbers of apoptotic cells, while miR-133a silencing promoted cell viability with fewer apoptotic cells (Fig. 2f, g).

### Silencing miR-133a promoted osteoblast differentiation in GC-treated MSCs

**Fig. 3:**
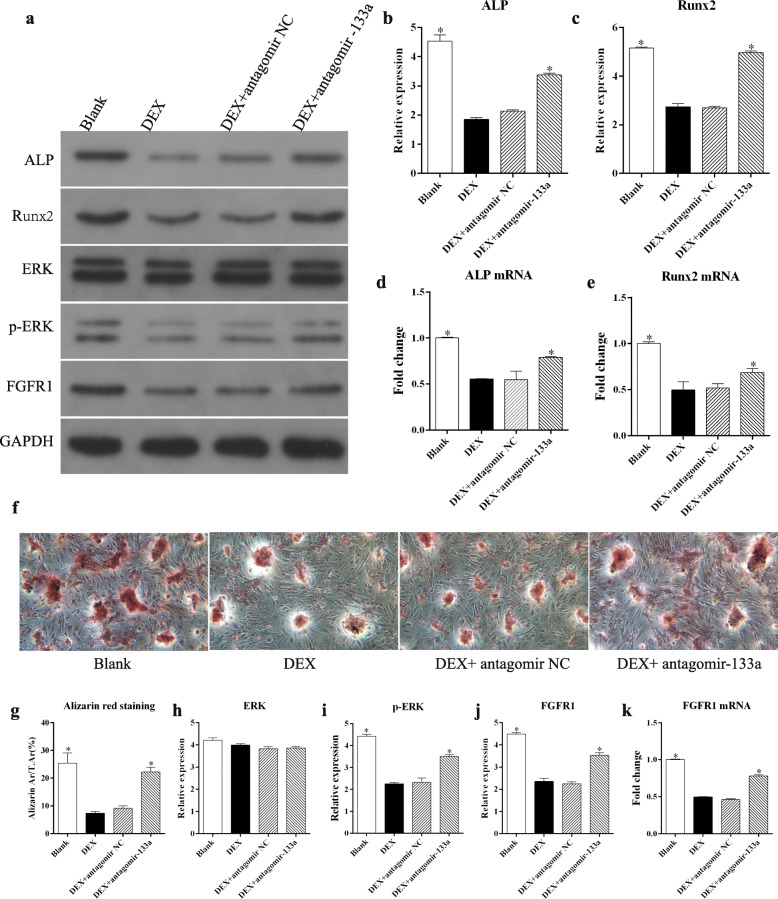
Silencing miR-133a promoted osteoblast differentiation in GC-treated MSCs. **a** Western blotting analysis for osteogenesis- and MAPK/ERK signaling-related proteins in MSCs after different treatments. **b**, **c** Gray analysis for the western blotting results of ALP and Runx2. **d**, **e** qRT PCR analysis of ALP and Runx2 mRNA in MSCs. **f** Representative images of alizarin red staining in MSCs after different treatments (× 100 magnification). **g** Quantification of alizarin red staining. **h**–**j** Gray analysis for the western blotting results of ERK1/2, p-ERK1/2, and FGFR1 proteins with osteogenesis induction medium. **k** qRT PCR analysis of FGFR1 mRNA in MSCs with osteogenesis induction medium (each condition was performed in triplicate, * *P* < 0.05 relative to the DEX group)

The miR-133a antagomir was transfected into MSCs to inhibit the expression of miR-133a, after which MSCs with or without the miR-133a antagomir were cultured in osteogenesis induction medium in the presence of DEX. Western blot analysis showed that DEX downregulated the expression of Runx2 and ALPs, indicating DEX suppression of osteoblast differentiation while silencing miR-133a significantly upregulated the levels of Runx2 and ALP (Fig. 3a–c). Similarly, both Runx2 and ALP mRNA levels were downregulated by DEX but upregulated by the miR-133a antagomir under the same conditions (Fig. 3d, e). The mineralization assay further demonstrated the inhibition of osteogenesis by DEX and its promotion by miR-133a silencing (Fig. 3f, g). To explore the mechanism of miR-133a silencing in MSC osteogenesis, we measured the phosphorylation of ERK1/2, key proteins in the MAPK/ERK signaling pathway. Western blot analysis showed that ERK phosphorylation was significantly decreased by DEX and significantly increased by the miR-133a antagomir (Fig. 3a, h, i). Meanwhile, FGFR1, an important regulator of ERK1/2 phosphorylation, was also downregulated by DEX and upregulated by the miR-133a antagomir (Fig. 3a, j, k).

### Silencing miR-133a inhibited adipocyte differentiation in GC-treated MSCs

**Fig. 4:**
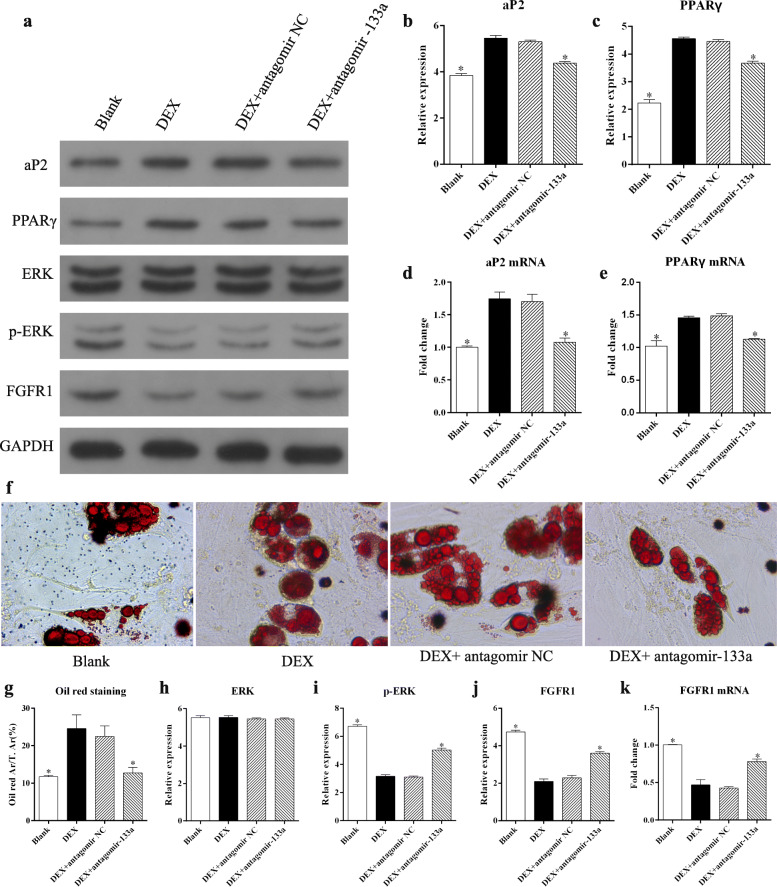
Silencing miR-133a inhibited adipocyte differentiation in GC-treated MSCs. **a** Western blotting analysis for adipogenesis- and MAPK/ERK signaling-related proteins in MSCs after different treatments. **b**, **c** Gray analysis for the western blotting results of aP2 and PPARγ. **d**, **e** qRT PCR analysis of aP2 and PPARγ mRNA in MSCs. **f** Representative images of oil red staining in MSCs after different treatments (×400 magnification). **g** Quantification of oil red staining. **h**–**j** Gray analysis for the western blotting results of ERK1/2, p-ERK1/2, and FGFR1 proteins in MSCs with adipogenesis induction medium. **k** qRT PCR analysis of FGFR1 mRNA in MSCs with adipogenesis induction medium (each condition was performed in triplicate, * *P* < 0.05 relative to the DEX group)

As the balance between osteogenesis and adipogenesis in MSCs plays an important role in the pathogenesis of GIO, we explored the effect of miR-133a silencing on adipocyte differentiation in GC-treated MSCs cultured in adipogenesis induction medium. The results indicated that while the expression of aP2 and PPARγ was significantly upregulated by DEX, miR-133a silencing resulted in a clear downregulation of these adipogenesis-related proteins (Fig. 4a–c). Similar results were obtained in the qRT-PCR analysis of the aP2 and PPARγ mRNA (Fig. 3d, e). Oil red staining indicated that DEX promoted adipocyte differentiation in MSCs, which could be attenuated by the miR-133a antagomir (Fig. 4, f, g). As in the osteogenic induction, DEX significantly suppressed both the phosphorylation of ERK1/2 and the levels of FGFR1, which could be rescued by the miR-133a antagomir (Fig. 4a, h–k).

### Silencing miR-133a abrogates bone mass loss in GIO rats

**Fig. 5:**
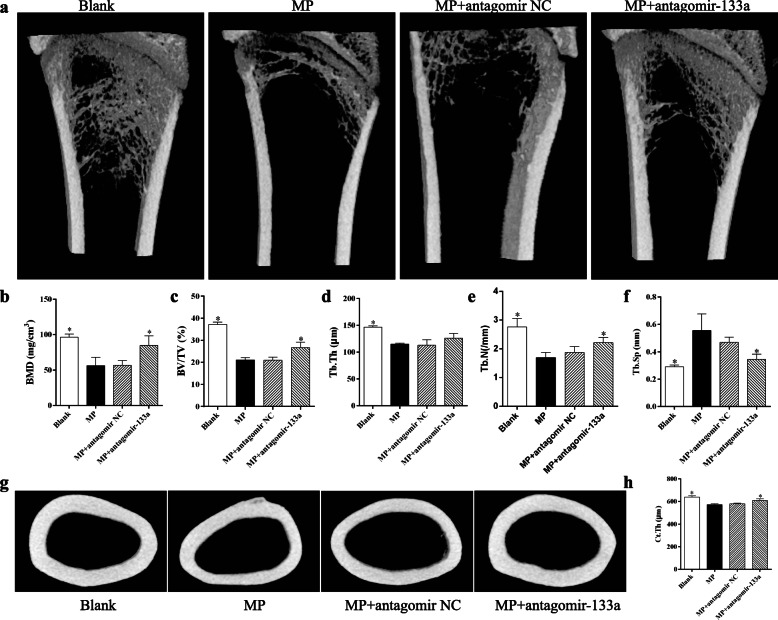
Silencing miR-133a abrogates bone mass loss in the distal and middle femurs of GIO rats. **a** Representative 3-D images of the distal femurs of rats in each group. **b**–**f** Analysis of trabecular parameters in distal femurs of rats from each group. **g** Representative 3-D images of the middle femur of rats from each group. **h** Analysis of cortical thickness in the middle femur of rats from each group (*P < 0.05 relative to the MP group)

Having shown that miR-133a silencing abrogated the effect of GC on MSCs and the upregulation of miR-133a in GC-treated rats, we investigated whether miR-133a silencing improves the bone architecture of GIO models. To this end, we developed a GIO rat model by injecting methylprednisolone and modulated the miR-133a expression in the femur by intramedullary injection of the miR-133a antagomir. The dose of methylprednisolone intramuscularly injection is not lethal to rats according to our previous observation, although the mortality rates in the subgroups were 2/10 in the Blank group, 1/10 in the MP group, 2/10 in the MP + antagomir NC group, and 1/10 in the MP + antagomir-133a group. The general anesthesia for intramedullary injection may contribute to this phenomenon in this study. Micro-CT analyses showed that, compared with the PBS-treated mice (Blank group), high doses of MP led to significantly decreased trabecular bone mineral density (BMD), trabecular bone volume/total volume (BV/TV), trabecular thickness (Tb. Th), and trabecular number (Tb. N), coupled with increased trabecular separation (Tb. Sp) in the distal femur (Fig. 5a–f). These results indicated the successful induction of the osteoporosis model in the rats. Meanwhile, the cortical thickness (Ct. Th) in the middle femur was also decreased by MP injection (Fig. 5g, h). However, miR-133a silencing by the miR-133a antagomir attenuated the loss of bone mass and improved the trabecular and cortical parameters induced by MP.

**Fig. 6:**
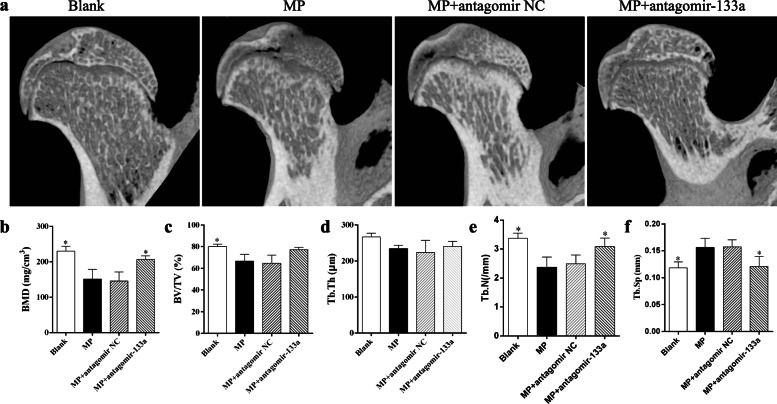
Silencing miR-133a abrogates bone mass loss in the femoral heads of GIO rats. **a** Representative 3-D images of the femoral heads from each group. **b–f** Analysis of trabecular parameters in femoral heads from each group (*P < 0.05 relative to the MP group)

Trabecular damage in the subchondral area of the femoral head is one of the pathological features of GC-induced osteonecrosis [16]. We further investigated the trabecular parameters of the femoral heads in rats of each group. The data showed similar results to those seen in the distal femur, with MP treatment inducing obvious bone mass loss in the subchondral area and the miR-133a antagomir attenuating this phenomenon, thereby improving the trabecular architectures (Fig. 6).

### Silencing miR-133a increases bone formation and decreases marrow fat accumulation in GIO rats

**Fig. 7:**
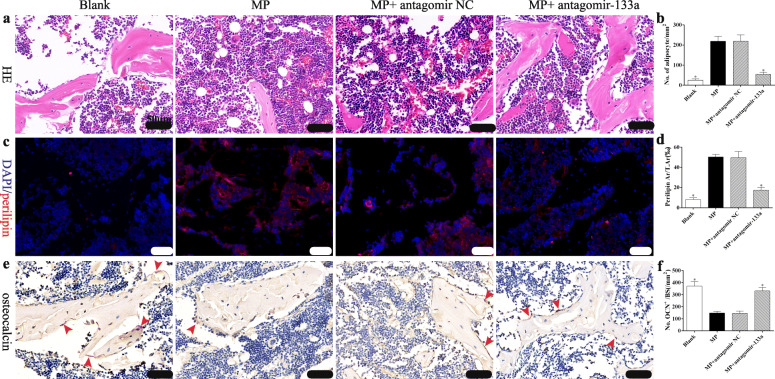
Silencing miR-133a attenuates marrow fat accumulation and trabecula damage in distal femurs of GIO rats. **a** Representative images of H&E staining showing trabecula and marrow adipocytes in distal femur of each group. **b** Quantification of number of adipocytes in distal femur of each group. **c**, **d** Representative images of immunofluorescence staining (**c**) and quantification of perilipin area /total area (T.Ar) in distal femur. **e**, **f** Representative images immunohistochemical staining (**e**, arrows indicate osteocalcin positive osteoblasts) and quantification of osteoblasts per bone surface (BS) in distal femur (scale bar 50 μm, *P < 0.05 relative to the MP group)

We next investigated whether miR-133a silencing attenuated bone mass loss in GIO rats by promoting bone formation or inhibiting bone marrow fat accumulation or both. H&E staining revealed sparser trabecula and more marrow fat accumulation in the distal femurs of rats in the MP group compared with those in the Blank group, while the miR-133a antagomir increased the number of trabecula and decreased the number of adipocytes in the marrow (Fig. 7a, b). Immunofluorescence staining for perilipin showed significantly increased numbers of bone marrow adipocytes in rats treated with MP only, similar to the rats in the MP+ antagomir NC group. Concomitantly, the miR-133a antagomir decreased the number of red staining cells in the marrow (Fig. 7c, d). Next, we investigated bone formation in the distal femur via osteocalcin immunochemical staining, which showed decreased numbers of osteoblasts on the trabecular and endosteal bone surfaces in the rats in the MP and MP+ antagomir NC groups compared to the Blank group, while higher numbers of osteoblasts were observed in the MP + miR-133a antagomir group (Fig. 7e, f). These data indicated that the miR-133a antagomir attenuated bone mass loss in GIO both by promoting bone formation and inhibiting bone marrow fat accumulation.

**Fig. 8:**
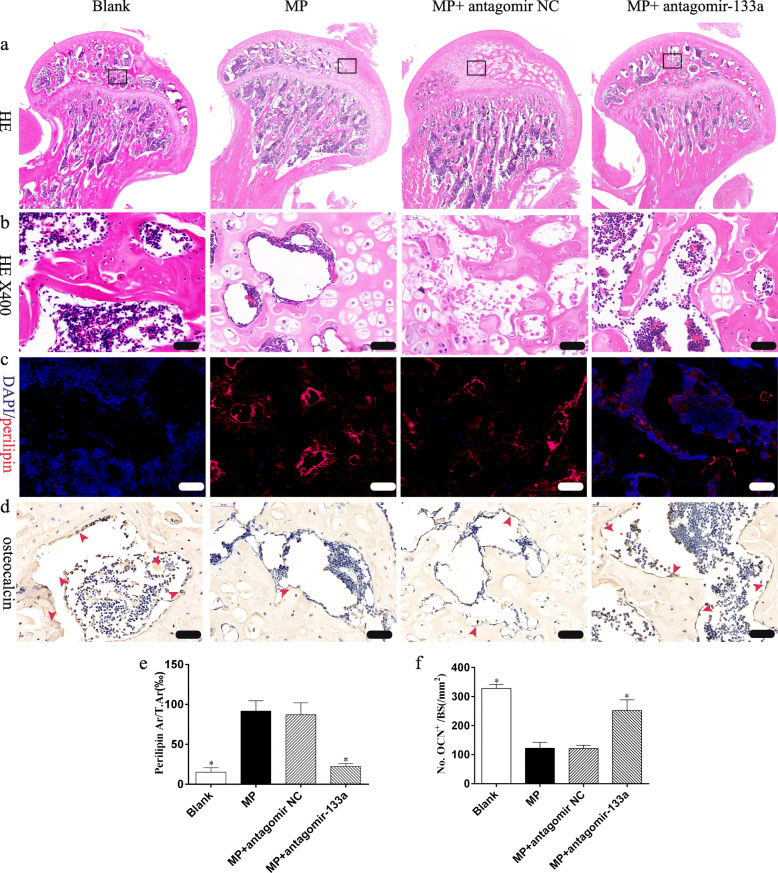
Silencing miR-133a attenuates marrow fat accumulation and trabecula damage in femoral heads of GIO rats. **a**, **b** Representative images of H&E staining in femoral heads of each group. **c** Representative images of immunofluorescence staining. **d** Representative images of immunohistochemical staining (arrows indicate osteocalcin positive osteoblasts). **e** Quantification of perilipin area/total area (T.Ar). **f** Quantification of osteoblasts per bone surface (BS) (scale bar 50 μm, *P < 0.05 relative to the MP group)

Similar results were obtained in the histological analysis of the femoral heads. Increased numbers of non-structural chondrocytes and decreased numbers of osteoblasts were observed in the femoral heads of rats treated with MP only, while the miR-133a antagomir decreased marrow fat accumulation and increased bone formation in GIO rats (Fig. 8).

## Discussion

Since MSC dysfunction plays an important role in the pathogenesis of GIO, MSC-targeting interventions may become a promising approach to treat or prevent glucocorticoid-induced osteoporosis. In this study, we demonstrated that miR-133a was strongly associated with GIO, especially with MSC disorders induced by glucocorticoids. Notably, silencing of miR-133a exerted positive effects on GC-treated MSCs, including the promotion of cell proliferation, cell viability, and the balance of osteoblast/adipocyte differentiation. All these factors resulted in the prevention of bone loss by local miR-133a silencing in rats with GIO. However, although the in vivo study appreciated a recovery in bone structure after silencing of miR-133a, some parameters measured in this study improved slightly, since many factors may influence bone formation and bone resorption in the process of osteoporosis.

miR-133a is a muscle-enriched microRNA that is involved in cardiac and skeletal muscle development [17, 18] and acts as a central upstream regulator of brown adipogenesis [19] and a suppressor in tumor growth and metastasis [20, 21]. The role of miR-133a in bone metabolism has also been reported in several studies. Du [22] demonstrated that miR-133a was downregulated during osteogenic induction of rat dental follicle cells while Plummer [23] observed an elevated expression of miR-133a in mice with weaker bones. Clinical studies have also demonstrated higher expression levels of miR-133a in patients with fracture nonunion [24] and patients with postmenopausal osteoporosis. Our study further illustrated that miR-133a was highly expressed in GC-treated rats which experienced obvious bone loss; local inhibition of miR-133a attenuated the bone loss and improved bone architecture in rats.

Most studies have found that the role of miR-133a in bone metabolism was related to the modulation of MSC osteoblast differentiation [12, 15, 25], which is in line with our findings. Furthermore, we demonstrated that MSC adipocyte differentiation was also involved in the regulatory effects of miR-133a on bone as silencing miR-133a protects MSCs against glucocorticoids both by promoting osteoblast differentiation and inhibiting adipocyte differentiation.

Runx2 and PPARγ are master regulators of MSC osteoblast and adipocyte differentiation, respectively, and the two transcription factors influence each other to achieve the differentiation balance [26]. It has been found that Runx2 is antagonized by glucocorticoids during MSC osteoblast differentiation [3] and that Runx2 mRNA was a direct target of miR-133a, which inhibits osteoblastic differentiation-associated markers [12]. In agreement with the previous studies, we also observed downregulation of Runx2 induced by DEX and upregulation by miR-133a silencing, indicating the antagonistic effects of miR-133a silencing on glucocorticoids. Moreover, we found that the expression of PPARγ and aP2 was upregulated by DEX, resulting in the promotion of MSC adipocyte differentiation, which is in line with the findings of previous studies [1, 5]. While the expression of PPARγ and aP2 was downregulated by miR-133a silencing, we speculate that the inhibition of adipogenesis is either associated with Runx2 or with a signaling pathway targeted by miRNA-133a.

The MAPK/ERK signaling pathway is known to be involved in the proliferation, viability, and differentiation of MSCs. Specifically, activation of MAPK/ERK signaling promotes cell proliferation and increases cell viability and defense against apoptosis [27, 28] while inactivation of this pathway has been found to block osteoblast differentiation and promote adipocyte differentiation in MSCs [29, 30]. As shown in this study, glucocorticoid significantly inhibited the phosphorylation of ERK in MSCs, a finding consistent with other studies and which may contribute to the deleterious effects of glucocorticoids on MSCs [31, 32]. Silencing miR-133a promoted ERK phosphorylation and attenuated the deleterious effects of the glucocorticoid, indicating that miR-133a may regulate GIO through the MAPK / ERK signaling pathway as well as through Runx2.

Fibroblast growth factor (FGF)/ (FGFR) signaling plays an important role in skeletal development, and multiple FGF/FGFRs have been characterized in MSCs [33, 34]. Studies have shown that FGFR1 signaling can stimulate MSC proliferation and enhance their viability [34]; moreover, activation of FGFR1 promotes the differentiation of mesenchymal progenitors into osteoblasts [33, 35]. Although the mechanisms of FGFR1 signaling are complex, the phosphorylation and activation of ERK have been shown to play major roles in the process [33, 35, 36]. Our study further illustrated the function of FGFR1/ERK signaling in MSCs, with high doses of glucocorticoid downregulating the expression of FGFR1 and subsequently attenuating the activation of MAPK/ERK signaling. Meanwhile, as a predicated target of miR-133a [37, 38], FGFR1 was strongly upregulated by miR-133a silencing in MSCs cultured with glucocorticoid, which further expands the role of miR-133a in GIO.

## Conclusion

To summarize, our findings suggest that miR-133a is strongly associated with GIO and similar disorders induced by glucocorticoids in MSCs. Silencing miR-133a resulted in positive effects on GC-treated MSCs and on bone loss in GIO animal models. Moreover, the FGFR1-MAPK/ERK signaling may be involved in the protective effect of miR-133a silencing.

## Data Availability

The datasets supporting the conclusions of this article are included within the article and could be obtained from the corresponding author if necessary.

## Abbreviations

ALP: Alkaline phosphatase
aP2: Adipocyte protein 2
BMD: Bone mineral density
BMP2: Bone morphogenetic protein 2
BSA: Bovine serum albumin
BV/TV: Bone volume per tissue volume
CCK-8: Cell Counting Kit-8
Ct. Th: Cortical thickness
DEX: Dexamethasone
DMEM: Dulbecco’s modified Eagle’s medium
ECL: Electrochemiluminescence
ERK1/2: Extracellular regulated protein kinase1/2
FBS: Fetal bovine serum
FGFR: Fibroblast growth factor receptor
GAPDH: Glyceraldehyde phosphate dehydrogenase
GC: Glucocorticoid
GIO: Glucocorticoid-induced osteoporosis
HRP: Horseradish peroxidase
MAPK/ERK: Mitogen-activated protein kinase/ extracellular regulated protein kinase
MP: Methylprednisolone
MSC: Mesenchymal stem cells
OCN: Osteocalcin
PBS: Phosphate buffer saline
p-ERK1/2: Phosphate extracellular regulated protein kinase1/2
PPARγ: Peroxisome proliferator-activated receptor γ
qRT-PCR: Quantitative real-time polymerase chain reaction
RUNX2: Runt-related transcription factor 2
Tb. N: Trabecular number
Tb. Sp: Trabecular separation
Tb. Th: Trabecular thickness
